# Telehealth for Addressing Sexual and Reproductive Health and Rights Needs During the COVID-19 Pandemic and Beyond: A Hybrid Telemedicine-Community Accompaniment Model for Abortion and Contraception Services in Pakistan

**DOI:** 10.3389/fgwh.2021.705262

**Published:** 2021-07-26

**Authors:** Irum Shaikh, Stephanie Andrea Küng, Hina Aziz, Samina Sabir, Ghulam Shabbir, Mukhtar Ahmed, Rasha Dabash

**Affiliations:** ^1^Ipas Pakistan, Islamabad, Pakistan; ^2^Ipas, Chapel Hill, NC, United States

**Keywords:** abortion services, sexual and reproductive health and rights (SRHR), COVID-19, telehealth, Pakistan, digital health, contraception, telemedicine

## Abstract

The COVID-19 pandemic led overburdened health care systems to deprioritize essential sexual and reproductive healthcare, including abortion and contraception care, while accelerating shifts in healthcare delivery to digital technologies. However, in many countries, including Pakistan, inequalities in access to digital technologies remain, presenting an opportunity for interventions that both increase access to deprioritized sexual and reproductive health and rights (SRHR) services and overcome the digital divide in delivering digital solutions to those in need of SRHR services. In June 2020, Ipas Pakistan partnered with Sehat Kahani (SK), a local health care NGO and telehealth service, and an existing network of Lady Health Workers (LHWs) to launch a novel hybrid telemedicine-community accompaniment pilot. The model linked women *via* LHWs with mobile devices to online providers for telemedicine consultations for SRH, including abortion services, contraception, and other gynecological consultations. In June 2020, we trained 98 LHWs and 22 telehealth doctors. Between June 2020 and March 2021, a total of 176 women were referred by LHWs for telehealth consultations. Among women who received abortion services, nearly all (90%) reported complete uterine evacuation. No serious adverse events were reported. Overall satisfaction was high; 81% reported being satisfied, and 86% said it is likely they would recommend the telehealth service to others. Data show that the provision of SRHR services *via* a telehealth-accompaniment model can be successfully implemented in Pakistan. Outcome data show high satisfaction and good clinical outcomes for women accessing care through this model. However, more data are needed to understand the full potential of this model. Barriers to digital health models, such as poor or inconsistent internet access, remain in places like Pakistan, especially in rural settings. This approach has its limitations but should be considered as an option in settings with similarly established community health networks and inequitable access to digital health.

## Introduction

The COVID-19 pandemic has had profound impacts on how healthcare is delivered and accessed, as well as what types of healthcare are considered essential. Around the world, overburdened health care systems deprioritized certain services in an effort to prioritize pandemic response. Sexual and reproductive healthcare, and in particular abortion and contraception, were seen as less essential health care services, leaving many people with no recourse for preventing or terminating pregnancies. Spurred by the absence of essential commodities for abortion and contraception, suspension of sexual and reproductive health services, lockdowns and reluctance to go to health facilities, and an increase in gender-based violence, it is estimated that the pandemic could result in an additional 49 million women without access to modern contraceptives and an additional 3.3 million unsafe abortions in low-to-middle-income countries (1). In many countries, anticipated impacts on access to abortion and contraception exacerbate existing barriers.

The COVID-19 pandemic also accelerated shifts in health care delivery and innovations that had already begun to seed, bringing healthcare, similar to so many other aspects of human life, to virtual platforms. This was particularly important and opportunistic where technologies and innovations are available and easy to use, like contraception and medical abortion (MA). However, some countries experienced these shifts more quickly and more seamlessly than others, where uneven phone and internet coverage, as well as inequitable distribution of devices and technological literacy make transitions to digital health care services untenable for all (2, 3).

Inequitable access to technology means that new digital solutions to health care delivery are difficult or impossible to implement in many settings, and may even exacerbate existing inequalities. In an effort to respond to the diminished access to clinic-based services during the pandemic while remaining cognizant of barriers to digital health solutions, Ipas Pakistan launched a novel hybrid telemedicine-community accompaniment pilot in June 2020. This model aimed to bring sexual and reproductive health and rights (SRHR) services typically provided in the clinic setting to women and girls in Pakistan *via* digital platforms by combining telemedicine with community accompaniment such that access to the Internet and technology was not a prerequisite to services. This paper presents the development and implementation of the Ipas's hybrid telemedicine-accompaniment model in Pakistan and some key lessons learned.

## Context

Pakistan, in South Asia, is the fifth most populous country in the world (4). It is defined by the World Bank as a lower middle income country (5) and was ranked 154 of 195 countries on health care access and quality (6). At the time of this writing, Pakistan had over half a million COVID-19 cases and over 16,600 deaths (7). Due to low testing rates and issues in reporting, these numbers are likely underreported.

Access to contraception and abortion services in Pakistan is limited; in 2012 an estimated 20% of women in Pakistan had an unmet need for contraception (8). Lack of widespread contraceptive access in the country also results in high rates of unintended and unplanned pregnancy, as well as abortion. Pakistan's abortion law was amended in 1997 to align with Islamic Principals; abortion is legal in Pakistan to save the person's life or to provide “necessary treatment” early in pregnancy, generally accepted by Islamic legal scholars as up to 120 days of pregnancy. However, a lack of clarity around the abortion law makes accessing safe abortion difficult. In 2012 almost 698,000 women were treated at health facilities for complications due to unsafe abortions in Pakistan, and unsafe abortion accounts for almost 6% of maternal deaths in the country (8).

Mobile phone usage in Pakistan is growing; approximately 47% of the population has a mobile phone. However, key components to facilitate widespread access (e.g., broadband coverage, infrastructure, and affordability) rank low in Pakistan when compared to its neighbors. In 2016, only 17% of Pakistanis owned a smartphone, and only 10% subscribed to mobile broadband services, although this was estimated to triple by 2020, largely driven by access to more affordable smartphones and improved network coverage (9). Nonetheless, Pakistan ranked 90 of 120 countries on the Inclusive Internet Index, and internet access remains unavailable and unreliable in particular in rural Pakistan (10). There are also important gender gaps in digital access in Pakistan; women are 37% less likely than men to own a mobile phone (11). As for digital literacy, 40% of mobile phone owners in Pakistan report difficulty understanding how to use their phones (9).

As Pakistan struggles to contain the COVID-19 pandemic, SRHR services remain essential but out of reach for many. However, gaps in mobile phone access, especially among gender lines, and internet connectivity make clear that a pure telehealth model for expanding SRHR services is not feasible in Pakistan. Our hybrid model attempts to make telehealth SRHR services available to women and girls by using community-based health workers with access to smart devices as intermediaries between women and doctors.

## Key Programmatic Elements: Ipas's Hybrid Telemedicine-Community Accompaniment Model

Starting in June 2020, Ipas Pakistan partnered with Sehat Kahani (SK), a local health care NGO, and the Punjab Department of Health to develop a hybrid abortion and contraception telemedicine model that provided women and girls a different way to access SRHR services that were not easily accessible during the pandemic. The model relies on both remote clinical consultation by doctors and in-person accompaniment by LHWs to overcome barriers of the digital divide and support women in their SRHR needs before, during, and after the remote consultation. It builds on the benefits of both digital technology and Pakistan's investments in community-based providers for continuity of care. The intervention was implemented in selected districts in Punjab (Jhelum, Chakwal, Sahiwal, and Multan) and the Federal capital territory of Islamabad. Target beneficiaries were women and girls of reproductive age, comprising approximately 2.8 million people. To assess the strength of the model, Ipas collected data on referrals through the SK app and qualitative feedback from LHWs throughout program implementation, as well as monitoring data among a sample of women 5–7 weeks after their telehealth consultation.

In June 2020, Ipas Pakistan virtually trained 16 gynecologists and general practitioners from the SK network and 6 general practitioners new to abortion and family planning care from public sector facilities. Providers were trained on safe abortion services, as well as family planning and post-abortion contraception. The training also included Values Clarification and Attitude Transformation (VCAT) workshops and covered updated guidance from the WHO on COVID-19, which includes specific guidance on safe abortion services and encourages the use of telehealth so that physical visits to health facilities could be minimized (12).

In parallel, Ipas Pakistan trained a total of 98 LHWs in Punjab province to provide accompaniment support and assist women in accessing a telemedicine consultation *via* SK's web-based application that connects women to a trained SRH provider. LWHs were trained to support women throughout the process and respond to questions and needs. Only LHWs who had a smartphone were selected for training for the pilot; these LHWs were provided with mobile data plans to enable use of the SK app. LHWs are a paid network of community-based health workers specifically trained to deliver a range of maternal and child health services, including family planning services and referrals, to women, girls, and children. All LHWs must undergo 15 months of training in family planning and primary healthcare to be qualified as an LHW (13). The LHWs selected for implementation of this model were all previously trained by Ipas and were well-versed on safe abortion methods, post abortion care, and referral linkages but were new to this model and their role providing accompaniment to women.

By establishing a network of trained telehealth clinicians as well as LHWs trained on the usage of the SK app, community members in need of abortion, contraception, or general gynecological consultations could approach LHWs in their community and, through the LHW, be connected to a trained provider for a telehealth consultation. Provision of abortion services *via* this model included the provision of prescriptions for MA, instructions on the use of the medications, information on warning signs, and consultations on family planning. We use abortion services as an umbrella term that includes induced and post-abortion care services. Women could choose to be connected to any of the SK providers (including those trained by Ipas and those not trained by Ipas) for an immediate consultation *via* a direct service link or through a scheduled telehealth consultation using the app, or for a telehealth consultation with Ipas-trained providers *via* phone outside of the SK app. Scheduled telehealth consultations outside of the app allows for the continued provision of services even where internet connection is disrupted or in the case of low digital literacy. For women needing abortion services, e-prescriptions for misoprostol were generated after the teleconsultation. While misoprostol is widely available in pharmacies in Pakistan at a low cost, a prescription is often required. LHWs also provided accompaniment to health facilities and/or pharmacies for MA medications, family planning methods, and follow-up after the telehealth service. This model benefits from an already established network of LHWs that are well known to community members. LHWs serve not only as a referral link to trained doctors, but also as a physical bridge to link community members with telehealth services, thus overcoming the digital divide and expanding the benefit of digital health services to those who need it.

## Methods

Ipas Pakistan collected data throughout the implementation of the hybrid telemedicine-accompaniment pilot in order to respond to three main questions: (1) is the hybrid model helping women and girls access contraception and abortion services during the COVID-19 pandemic in Pakistan? (2) does this model support capacity building of community intermediaries on the use of digital technology for connecting community women to clinical consultations and prescriptions for abortion services?; and (3) is a telehealth method for delivering abortion services acceptable among women in Pakistan? Data are included from three different sources: (1) referral data from LHWs from the SK app database, (2) follow-up data collected by LHWs 5–7 days after telehealth consultations, and (3) feedback from LHWs. All data were considered monitoring data for program improvement and were not submitted for IRB approval.

Referral data were collected throughout the program implementation through the SK app database, which collects information on all referrals to the telehealth app, the mode of the telehealth consultation (direct instant link or a scheduled appointment), whether the consultation was completed, missed, or canceled, and the reason for the consultation. SK maintains this database and owns the data, and de-identified data were shared with Ipas on a monthly basis. Only the research team had access to the data, and data were kept in password-protected folders.

Additionally, from March to April 2021, Ipas Pakistan collected monitoring data from participants of the hybrid telehealth-accompaniment model in an effort to better understand clinical outcomes and general satisfaction with the model. All women who had been referred for telehealth consultations by LHWs were contacted. A verbal consent for participation was obtained and those who consented to participate were asked questions about the service they received, including recommended treatment, outcome of treatment, and satisfaction with the telehealth service. Data were collected by the LHWs who served as the referral to telehealth clinicians *via* phone or in person, as these LHWs had existing relationships and extensive contact with the women. LHWs collected responses using a paper-based questionnaire, which were then shared with Ipas in person *via* sealed envelopes. Members of the research team then entered the data in Excel for further analysis. Data were kept in password-protected folders.

Finally, feedback was regularly provided to Ipas staff during follow-up with LHWs in the field to understand the successes and continued challenges of this model. LHWs also relay perspectives shared by participants of the hybrid telehealth-accompaniment model. Perspectives of the LHWs themselves and of participants of the model were captured by Ipas staff for program improvement.

## Results

Between June 2020 and March 2021, a total of 176 women were referred by LHWs for telehealth consultations *via* the SK app (Table 1). A majority of clients (121; 69%) were connected directly to a provider using the direct instant link feature of the SK app, while 55 clients (31%) made telehealth appointments using the SK app. Nearly half (47%) of the consultations were completed, while 26% were canceled and 28% were missed. LHW referrals yielded 34 consultations for abortion services and counseling, and 31 consultations for gynecological and other general complaints (including vaginal discharge, antenatal and post-natal consultations, irregular menstrual complaints, and urinary tract infections). Sixteen clients received contraceptive counseling *via* telehealth consultations and providers issued three referrals for further consultation at a hospital.

**Table 1:** Referrals by LHWs *via* the SK app (*n* = 176), June 2020–March 2021.

	***N* (%)**
Total referrals by LHWs for telehealth consultations	176
Mode of consultation	
Direct instant link via SK app	121 (69%)
Appointment via SK app	55 (31%)
Status of consultation	
Completed	82 (47%)
Canceled	45 (26%)
Missed	49 (28%)
Reason for consultation, among those attended^a^	
Abortion consultation and counseling	34
Gynecological/other general complaints	29
Contraceptive counseling	16
Referral to hospital for further consultation	3

a *Due to the fact that one client can receive consultation on any number of issues, percentages cannot be calculated for this variable*.

A total of 88 women consented to providing monitoring data (Table 2). Average age of women who received telehealth services and provided monitoring data was 29, with ages ranging from 18 to 46 years. Sixty-two participants (70%) received consultations *via* the SK app and 26 (30%) made future appointments with Ipas-trained telehealth clinicians outside of the app. Nearly half of the women who reported monitoring data (42; 48%) received abortion services, 30 (34%) received consultations for contraception or other general gynecological consults, and 16 (18%) were referred to a facility for further consultation. All women who received abortion counseling services reported pregnancies of <13 weeks gestation. All were prescribed misoprostol and received post-abortion family planning (PAFP) counseling. Thirty-one women who received abortion services (74%) reported that they were also referred to a facility for an ultrasound. The majority of women (83%) receiving abortion services reported receiving the MA medication from a nearby clinic or hospital, while 4 (10%) got the pills from a pharmacy, and 3 (7%) from a community health worker. Most of them (74%) reported no issues taking the medication as prescribed and nearly all (90%) reported complete uterine evacuation. Women reported some side effects from the MA medication, but no serious adverse events were reported. The most frequently cited side effect was headache, followed by abdominal pain, fever, bleeding, and diarrhea. Overall satisfaction with the model was high; out of all 88 women, 71 (81%) reported being satisfied, 15 (17%) neutral, and 2 (2%) were unsatisfied. A large majority (86%) said it is likely that they would recommend the telehealth service to others, while 14% said recommendation is unlikely. Satisfaction and likelihood of recommendation was slightly lower among abortion clients when compared to all clients (71 *vs*. 81% and 76 *vs*. 86%, respectively).

**Table 2:** Monitoring Data (*n* = 88).

	**Abortion clients**	**Non-abortion clients**	**Overall**
	***n* = 42**	***n* = 46**	***n* = 88**
	***n*** **(%)**	***n*** **(%)**	***n*** **(%)**
Median age *(range)*	29 *18–46*	30 *18–40*	29 *18–46*
Reason for consultation			
Abortion counseling (including misoprostol prescription + PAFP counseling services)	42 (100%)	-	42 (48%)
Family planning counseling	-	13 (28%)	13 (15%)
Other gynecological consultations	-	17 (37%)	17 (19%)
Referral to hospital for further consultation	-	16 (35%)	16 (18%)
Mode of consultation			
Sehat Kahani app (via direct instant link or telehealth appointment)	34 (81%)	28 (61%)	62 (70%)
Telehealth consultations (to Ipas-trained providers *via* phone)	8 (19%)	18 (39%)	26 (30%)
Source of misoprostol, among abortion clients (*n* = 42)			
Pharmacy	4 (10%)	-	-
Nearby clinic/hospital	35 (83%)	-	-
Community health worker	3 (7%)	-	-
Able to take medication as prescribed, among abortion clients (*n* = 42)			
Yes	31 (74%)	-	-
No	11 (26%)	-	-
Uterine evacuation complete, among abortion clients (*n* = 42)			
Yes	38 (90%)	-	-
No	3 (7%)	-	-
Missing	1 (2%)	-	-
Side effects reported, among abortion clients (*n* = 42)^a^			
Abdominal pain	4	-	-
Diarrhea	1	-	-
Headache	8	-	-
Fever	4	-	-
Bleeding	3	-	-
Level of satisfaction with telehealth service			
Satisfied	30 (71%)	41 (89%)	71 (81%)
Neutral	11 (26%)	4 (9%)	15 (17%)
Unsatisfied	1 (2%)	1 (2%)	2 (2%)
Likelihood of recommendation of telehealth service to others			
Likely	32 (76%)	44 (96%)	76 (86%)
Unlikely	10 (24%)	2 (4%)	12 (14%)

*PAFP, post-abortion family planning*.

a *Due to the fact that one client could report multiple side effects, percentages cannot be calculated for this variable*.

In conversations with LHWs in the field, the model was seen as a great option for linking women to SRHR services, particularly during the COVID-19 pandemic. As one LHW shared,

“*We were reluctant to refer our patients to facilities due to COVID-19. This online system has helped us re-establish community linkages.”* (LHW)

This sentiment was shared by recipients of the model, as demonstrated in the following quotes:

“*I needed to see a doctor but was scared of visiting facility due to COVID-19, the lady health worker of my area told me about Sehat Kahani and linked me to a provider through app…the experience was amazing, I got connected to a provider who listened to me and gave detailed advice. It should continue and be available to every woman*.” (Telehealth client)

“*I had been visiting doctors in this COVID situation…by talking to a doctor with privacy and in the comfort of home, [it] felt like a blessing for me”* (Telehealth client)

## Discussion

Although numbers from this pilot program are small, our data show that this is a promising intervention for women and girls needing a different modality for accessing SRHR services in places where access to the Internet, technology, and in-clinic services is limited. We had sought to understand, first, if the hybrid model is helping women and girls to access SRHR services during the COVID-19 pandemic in Pakistan. With 176 referrals and a variety of consultations and services provided, including abortion services and contraception, this model has linked women to essential SRHR services during the COVID-19 pandemic in Pakistan.

Second, we asked whether this model supports the capacity building of community intermediaries on the use of digital technology for connecting community women to clinical consultations and prescriptions for abortion services. Trained LHWs successfully managed to connect community women to health care providers using the SK app and *via* phone to Ipas-trained providers, connecting at least 42 women to abortion services and at least 46 women to contraception services or other consults.

Finally, we asked whether a telehealth method for delivering abortion services is acceptable among women in Pakistan. Our monitoring data show high levels of reported satisfaction, high completion rates with MA, and no reports of adverse events or morbidity diagnoses. These results demonstrate the feasibility of using an existing network of LHWs to connect women with telehealth services; however, more data are needed on reasons behind high rates of incomplete consultations. Importantly, qualitative feedback from clients and LHWs shows that this model was particularly important to clients and community health workers during the COVID-19 pandemic, ensuring SRHR services remained available and accessible even as services were deprioritized in the clinic setting.

However, our data also show that there are many areas for improvement. For one, our monitoring data show that one in four women receiving consultations for abortion services reported some issues taking MA medication as prescribed, but data are lacking on why this is; more information is needed to understand barriers to taking medication as prescribed. Additionally, our data show that a high percentage of women did receive referrals for facility visits and went to a clinic or hospital to get the MA medication, despite the broad availability of misoprostol in pharmacies. In general, high linkage to clinics may be explained by the fact that LHWs are considered an extension of the clinics in communities, and/or due to restrictions on LHWs prescribing medication and lack of confidence in facilitating the entire service with no in-person physician oversight. High referrals for ultrasounds in the case of abortion services are likely due to discomfort relying on pregnant women for gestational age estimation particularly among providers new to abortion care and/or providers' own lack of confidence in gestational age estimation without an ultrasound. High referral rates to facilities may also help to explain why most women in monitoring data reported getting MA medication from the clinic or hospital, as many of these women may already have been at the facility for an ultrasound. However, more data are needed to understand the provider's high recourse to ultrasound for eligibility determination when not routinely required for MA, especially given concerns around accessing in-person services during the COVID-19 pandemic. Facility referrals should be supplemental to our telehealth-accompaniment model; requiring facility visits could reinforce barriers to SRHR access during the pandemic and weaken the value of this model. We plan to conduct additional training of providers and capacity building of LHWs around gestational age estimation to decrease unnecessary facility referrals and strengthen links to pharmacies for misoprostol.

Although this hybrid model may help to alleviate barriers caused by difficulties with digital literacy, access to mobile phones, and gender gaps in digital access, structural barriers remain when it comes to poor and inconsistent internet connectivity in the country. Problems with internet connectivity were made evident by frustrations shared by LHWs, especially in rural areas. As one LHW said, “coverage is poor, 3G is very slow…we face challenges in accessing Internet.”

LHWs are also, first and foremost, community health workers who provide basic health services to women, girls, and children. Some of the LHWs who participated in this pilot expressed difficulties managing their roles as community health providers *vs*. their roles in providing accompaniment. Facilitating balancing these two roles will be an important focus during project scale-up. Finally, women and LHWs both acknowledged challenges with finding available doctors *via* the app, especially considering not all SK providers are Ipas trained. In the case where no Ipas-trained providers were available, women could wait for one to become available through the app or could make a future appointment *via* phone. Ipas is working to expand the pool of trained telehealth clinicians through more virtual trainings.

It is also important to note that telehealth models are one pathway to care and should not be perceived as a magic bullet to access or siloed from the broader health system. MA is critical for expanding access to abortion services largely *because* people seeking abortion can do so on their own, with proven high efficacy and safety (14–17). This benefit cannot be overstated especially during the COVID-19 pandemic. The WHO acknowledges that shortages in health workers necessitate self-care interventions that enable individuals, families, and communities to promote health and manage illness with or without the support of a health care provider, but recommends that “self-care interventions must be an adjunct to, rather than a replacement for, direct interaction with the health system” (18). Existing accompaniment models, as well as this telehealth model, can provide guidance and support to people self-managing their abortions. However, linkages to providers trained in abortion care remain important, especially in the Pakistan context where LHWs are not legally allowed to prescribe the full range of contraceptive methods or MA drugs, such as misoprostol, but often have misoprostol on hand for the prevention of post-partum hemorrhage. This raises the question of whether there is potential for LHWs to handle the majority of abortion consultations on their own while connecting with providers in more complicated cases; such hotline and accompaniment models have been successful in other settings (19, 20). This would presumably require greater recognition and authorization for LHWs to formally take on this role, including supporting dispensing misoprostol which they already have access to for preventing post-partum hemorrhage (21, 22).

While our findings cannot be generalized to other settings, this model holds promise in expanding access to SRHR services in other countries and regions with inequitable access to digital health. Many countries rely on established networks of community health workers to provide health services, including Brazil, Ethiopia, Mozambique, Bangladesh, and Liberia (23–26). We urge groups working in such settings to consider implementing similar models.

## Conceptual or Methodological Constraints

Due to the urgency in the provision of remote SRHR services in Pakistan during the COVID-19 pandemic, one consolidated data capture system was not used during the initial phases of this pilot. As such, the findings presented here are from three different sources and may not reflect the true reach of this project. LHW referral data in particular suffered from missing data, especially regarding the different reasons for consultations, which limits our ability to assess SRHR needs during the COVID-19 pandemic as well as the true use of this model for abortion and contraception services and counseling. As not all who participated in this model provided monitoring data, it is possible that those who chose to respond had more positive experiences with the model. All monitoring data are self-reported and data on side effects and success of uterine evacuation may not have been clinically corroborated. In addition, respondents captured in monitoring data may have affiliated the LHWs who collected these data with the telehealth service, resulting in response bias, especially when asked about their satisfaction and likelihood of recommending the service. Nonetheless, monitoring data generated constructive feedback from clients.

Abortion remains stigmatized in Pakistan, and many clinicians fear that availability of abortion services may increase abortions rather than decrease unsafe abortions. This fear is even more pronounced when consultations are provided remotely. By engaging telehealth clinicians in multiple VCAT sessions and trainings and enlisting LHWs in gestational age dating, we hope to mitigate the stigma associated with abortion and increase confidence in client gestational age estimation to limit referrals to health facilities for ultrasounds. However, in order to scale up this initiative, these concerns, likely linked to fears of encountering legal problems, must be addressed.

The number of telehealth platforms has vastly increased in Pakistan during COVID-19 (e.g., PIMS Online Doctor, DoctHERs, Aman Telehealth, and Shifa for You); however, there is ambiguity around regulations and no clear policy especially in authorization of teleconsultation and e-prescription authentication. Ipas Pakistan is working with the Ministry of Health to establish clear protocols. Our model makes evident that telehealth platforms can and should include SRHR services, especially given the safety and ease of MA.

Finally, the poor or intermittent internet access in some areas of Pakistan may mean that this model cannot be feasibly implemented throughout the country. While a principal objective of this model is to bridge the digital divide created in part by inequitable access to the Internet, the success of the model is limited when LHWs themselves cannot access the Internet. Although the scheduling of phone appointments with trained providers outside of the SK app helps to alleviate the need for consistent internet access, technological advancements and infrastructure improvements are long-term investments that would help to expand the reach and success of this model. Accompaniment through additional means that do not require consistent internet access, such as messaging, may be future avenues for exploration.

## Contribution to the Field

The COVID-19 pandemic made clear the promise of digital health for provision of essential health services, especially as certain services were deprioritized and as people began avoiding in-person health care visits. However, such digital solutions, which include telemedicine, require access to mobile devices and internet access. In many countries, including Pakistan, access to mobile phones and reliable Internet is lacking, especially among women and in rural settings. This study shares findings from pilot implementation of a hybrid telemedicine-community accompaniment model for sexual and reproductive health services, including abortion and contraception services, in Pakistan.

Our model connected community members with trained LHWs who, equipped with a mobile phone and a telehealth app, were able to connect these women to a telehealth consultation with a trained clinician. Our data show promising preliminary results in being able to overcome the digital divide in provision of sexual and reproductive health services *via* a telehealth-accompaniment model. Outcome data show high satisfaction and good clinical outcomes for women accessing care through this model. This approach should be considered in settings with similarly established community health networks and inequitable access to digital health.

## Data Availability Statement

The datasets presented in this article are not readily available because the data analyzed for this study are sensitive in nature. The data are available from the corresponding author on reasonable request. Requests to access the datasets should be directed to Irum Shaikh, ShaikhI@ipas.org.

## Ethics Statement

Ethical review and approval was not required for the study on human participants in accordance with the local legislation and institutional requirements. Written informed consent for participation was not required for this study in accordance with the national legislation and the institutional requirements.

## Author Contributions

IS, RD, GS, SS, and MA were responsible for the conception, design, and implementation of the intervention. HA, SS, and MA acquired and analyzed the data. HA and SK interpreted the data. SK and IS wrote the first draft of the manuscript. HA, RD, GS, and SS provided critical review of the manuscript. All authors have given their final approval for publication.

## Conflict of Interest

The authors declare that the research was conducted in the absence of any commercial or financial relationships that could be construed as a potential conflict of interest.

## Publisher's Note

All claims expressed in this article are solely those of the authors and do not necessarily represent those of their affiliated organizations, or those of the publisher, the editors and the reviewers. Any product that may be evaluated in this article, or claim that may be made by its manufacturer, is not guaranteed or endorsed by the publisher.
